# Measuring the bias, precision, accuracy, and validity of self-reported height and weight in assessing overweight and obesity status among adolescents using a surveillance system

**DOI:** 10.1186/1479-5868-12-S1-S2

**Published:** 2015-07-27

**Authors:** Adriana Pérez, Kelley Pettee Gabriel, Eileen K Nehme, Dorothy J Mandell, Deanna M Hoelscher

**Affiliations:** 1 Michael & Susan Dell Center for Healthy Living, The University of Texas School of Public Health Austin Regional Campus, 1616 Guadalupe St., Austin, TX 78701, USA; 2 Department of Biostatistics, The University of Texas Health Science Center at Houston, (UTHEALTH), School of Public Health, 1616 Guadalupe, Suite 6.300, Austin, TX, 78701, USA; 3 Department of Epidemiology, Human Genetics and Environmental Sciences, The University of Texas Health Science Center at Houston(UTHEALTH), School of Public Health, 1616 Guadalupe, Ssuite 6.300, Austin, TX, 78701, USA; 4 Medical Research Specialist, Office of Program Decision Support, Family & Community Health Services, Texas Department of State Health Services, MC 1922, 1100 W. 49TH Street, Austin, TX 78756, USA; 5 Department of Health Promotion and Behavioral Sciences, the University of Texas Health Science Center at Houston(UTHEALTH), School of Public Health, 1616 Guadalupe, Suite 6.300, Austin, TX, 78701, USA

**Keywords:** children, youth, body mass index, correction equations, weighted linear regression

## Abstract

**Background:**

Evidence regarding bias, precision, and accuracy in adolescent self-reported height and weight across demographic subpopulations is lacking. The bias, precision, and accuracy of adolescent self-reported height and weight across subpopulations were examined using a large, diverse and representative sample of adolescents. A second objective was to develop correction equations for self-reported height and weight to provide more accurate estimates of body mass index (BMI) and weight status.

**Methods:**

A total of 24,221 students from 8th and 11th grade in Texas participated in the School Physical Activity and Nutrition (SPAN) surveillance system in years 2000–2002 and 2004–2005. To assess bias, the differences between the self-reported and objective measures, for height and weight were estimated. To assess precision and accuracy, the Lin’s concordance correlation coefficient was used. BMI was estimated for self-reported and objective measures. The prevalence of students’ weight status was estimated using self-reported and objective measures; absolute (bias) and relative error (relative bias) were assessed subsequently. Correction equations for sex and race/ethnicity subpopulations were developed to estimate objective measures of height, weight and BMI from self-reported measures using weighted linear regression. Sensitivity, specificity and positive predictive values of weight status classification using self-reported measures and correction equations are assessed by sex and grade.

**Results:**

Students in 8^th^- and 11^th^-grade overestimated their height from 0.68cm (White girls) to 2.02 cm (African-American boys), and underestimated their weight from 0.4 kg (Hispanic girls) to 0.98 kg (African-American girls). The differences in self-reported versus objectively-measured height and weight resulted in underestimation of BMI ranging from -0.23 kg/m^2^ (White boys) to -0.7 kg/m^2^ (African-American girls). The sensitivity of self-reported measures to classify weight status as obese was 70.8% and 81.9% for 8^th^- and 11^th^-graders, respectively. These estimates increased when using the correction equations to 77.4% and 84.4% for 8^th^- and 11^th^-graders, respectively.

**Conclusions:**

When direct measurement is not practical, self-reported measurements provide a reliable proxy measure across grade, sex and race/ethnicity subpopulations of adolescents. Correction equations increase the sensitivity of self-report measures to identify prevalence of overall overweight/obesity status.

## Background

Body mass index (BMI) is the most commonly used method to estimate overweight and obesity in children and adolescents, using standardized classification criteria based on the child’s height, weight, sex, and age [1-3]. BMI is often a critical variable included in worldwide surveillance systems and interventions to document outcomes of a program or policy, to describe epidemiology (i.e., person, place, and time) of childhood obesity, and/or to quantify the magnitude of obesity status within and across populations. In surveillance systems and interventions that include large and/or population-based sample sizes, adolescents’ height and weight are often obtained via self-report due to its low cost, ease of data collection, and the ability to efficiently collect data from a large number of individuals [4-6].

Some surveillance systems and other population-based studies of children and adolescents, including the National Longitudinal Study of Adolescent Health (U.S.), have incorporated ancillary studies where either all, or a subset of, participants’ heights and weights were directly measured and compared with self-reported estimates to examine validity. These comparison studies have been done in the U.S. [5-7], Wales [8], Portugal [9], Germany [10,11], and Australia [12]. In general, results of these studies have shown that, while adolescent-reported estimates of height and weight are correlated with objective measurements, they typically generate a lower estimate of overweight and obesity prevalence [6,7,13-16]

Some differences in the validity of self-reported height and weight data, by age or other socio-demographic factors, are well established. For example, studies have generally shown limited accuracy of self-reported height and weight among children aged younger than fourteen years [4,6,17,18]. Further, self-reported height and weight collected from girls tends to result in greater BMI underestimation than self-reported height and weight from boys [5,7,10,14,16,19-22]. The relatively few studies that have investigated differences by race/ethnicity have not yielded consistent results [7,13,14,16,23]. A few studies targeting specific ethnic subpopulations have also been conducted, including studies of Mexican Americans [24] and American Indians [15,25]. Despite numerous studies assessing validity of child-reported height and weight, gaps in understanding remain, particularly in regard to differences across subpopulations. A 2007 review of studies assessing the accuracy of self-reported height and weight in adolescents identified the lack of understanding about subpopulation differences as the primary gap in the literature on this subject [26]. To date, this gap has not been fully addressed.

The primary goal of this study was to examine, by subpopulations, the precision and accuracy of self-reported height and weight compared to objective measures of height and weight, in addition to the diagnostic validity of weight status (e.g., assumed objective measures as gold standard for estimating overweight and obesity), among a large, diverse and representative population of 8th- and 11th-grade adolescents in Texas, USA. Additionally, since population-based or intervention research often necessitates the collection of self-reported data, a secondary objective was to develop correction equations to estimate height, weight and BMI from self-reported height and weight data. These estimates could be used in lieu of objective measurement and improve the usefulness of self-reported measures in obesity prevention and intervention studies.

## Methods

The School Physical Activity and Nutrition (SPAN) project was designed to establish a surveillance system to monitor the prevalence of overweight and obesity among Texas school children in grades 4, 8 and 11. The description and design of SPAN has been previously reported [27-29]. Briefly, the first statewide SPAN survey was conducted over two academic years: 2000–2001 and 2001–2002, while the second statewide survey was administered in 2004–2005. SPAN utilized a sampling strategy involving nine Texas Health Service Region (HSR) levels, three types of communities (urban center, other urban/suburban and rural) and three grade levels, to yield representative data at the Texas state, Texas Health Service Region (HSR) levels, and for three major racial/ethnic groups in Texas**:** African-American, Hispanic and white/other. Unfortunately, other races/ethnicities were not considered as a subpopulation due to the low prevalence in Texas and large sample size needed to make a representative sample of other race/ethnicities. The sampling frame was created based on school and school district-level data made available from the Texas Education Agency (TEA) from the academic year preceding each respective SPAN survey. Sampling weights and post-stratification adjustments accounted for the complex design, differential representation, the use of stratification and sampling clusters, as well as updates in the sampling frame for each survey administration [27-29]. The SPAN survey included items to assess (1) demographic characteristics (e.g., sex, grade, and race/ethnicity); (2) dietary intake, including meal patterns and nutrition knowledge; (3) physical activity; and (4) reported height and weight. The SPAN survey instruments for grades 8 and 11 are identical and have been previously shown to be valid and reliable [30,31].

The first administration of the statewide SPAN survey included a sample of 5,362 and 3,576 8th- and 11th-grade children, respectively. This sample was representative of a population of 288,584 and 249,363 8th- and 11th-grade children, respectively. The second statewide SPAN survey included a sample of 8,827 and 6,456 8th- and 11th-grade children, respectively, representing their respective grade populations of 291,672 and 233,753 students.

## Human subjects and consent procedures

Approval for this study was obtained from (1) the Committee for the Protection of Human Subjects at The University of Texas Health Science Center at Houston (HSC-SPH-00-056), (2) the institutional review board of the Texas Department of State Health Services (04-062) and (3) participating school districts. Depending on the school or school district, parental consent was obtained via either active or passive methods, and study participants (i.e., children) provided assent prior to data collection.

## Measures

### Demographic characteristics

Demographic variables collected include sex, age, grade, and race/ethnicity. Categories of response for self-reported race/ethnicity were: Black or African-American; Mexican-American, Latino or Hispanic; White, non-Hispanic, non-Latino; American Indian or Alaska Native; Asian; Native Hawaiian or Other Pacific Islander; White, non-Hispanic, non-Latino; and Other. These were collapsed into three main race/ethnicities: African-American, Hispanic, or White/other. For international comparison purposes, in the U.S., children begin their first year of formal education (kindergarten) at age 5. Eighth grade is the ninth year of formal education, also known as the third year of middle school, or lower secondary education (level 2) as classified by the United Nations Educational, Scientific and Cultural Organization’s International Standard Classification of Education (ISCED) [32]. Similarly, in the U.S., 11th grade is the twelfth year of formal education, also known as the third year of U.S. high school, or upper secondary education (ISCED level 3). As in many countries worldwide [33], U.S. students typically begin 8^th^-grade at age 13 years and 11^th^-grade at age 16 years.

### Self-reported measures of height and weight

Self-reported height, recorded in feet and inches, was converted to centimeters and self-reported weight, recorded in pounds, was converted to kilograms to standardize units of expression for comparison with the objective measures of height and weight. Self-reported height (without shoes) and weight (without heavy clothes and shoes) data were collected from students in 8^th^- and 11^th^-grade. These grades were chosen in line with recommendations to not collect these measures from 4^th^-grade children (aged approximately 9-10 years) due to their general inability to give accurate or reasonable values for height or weight [4,6,17].

### Objective measures of height and weight

Students’ heights and weights were measured using standardized procedures. Children removed any heavy clothes and shoes before having their height and weight measured. Height was measured to the nearest 0.1 centimeter with a portable stadiometer (Perspective Enterprises Portable Adult Measuring Unit PE-AIM-101) and weight was measured to the nearest 0.1 kg with a portable digital scale with remote display (Tanita Professional Digital Scales with Remote Display, BWB-800S) calibrated to 113 kg (i.e. 250 pounds) before each series of measurements. Study staff recorded both measures on the student questionnaires.

Using both the self-reported and objective measures, BMI was computed as weight (kilograms) divided by height (meters) squared. Then, both BMI estimates (self-reported and objective measures) were collapsed to categories reflecting weight status (i.e., underweight/normal (<85th percentile), overweight (≥85th percentile to <95th percentile) and obese (≥95th percentile)) using the Centers for Disease Control and Prevention growth charts [34,35].

## Statistical analyses

All statistical analysis for estimation takes the form of weighted statistics, using the sampling weights from each statewide survey. This provides the opportunity to look at differences in reported height and weight by (1) sex, (2) race/ethnicity, and (3) grade level. SPAN did not sample students by age, which precludes the ability to present and examine the parameters of interest by age. Differences between the self-reported and objectively-measured height and weight were explored between the two administrations of the SPAN survey (i.e., 2000–2001 versus 2004–2005 academic years) using weighted regression analysis. There were no statistically significantly differences between the first and second SPAN surveys in the self-reported and objectively-measured data adjusting by sex, grade, and race/ethnicity. Therefore, data were pooled to enhance statistical power.

Second, descriptive statistics including (1) age (mean ± standard error), (2) self-reported and objectively-measured BMI (including height and weight estimates; median ± standard error), and (3) proportion within sex, race/ethnicity, and weight status categories (% ± standard error) were calculated for each statewide survey separately and then pooled across both survey administrations. Median values with 95% confidence intervals (CI) of height, weight, and BMI were reported due to lack of normality. Third, weight status prevalence estimates, using self-report and objectively-measured estimates were reported by (1) grade and (2) sex. Then, the absolute error (bias), calculated as the difference between the self-reported prevalence and the objectively-measured prevalence, and relative error, calculated as the absolute error divided by the objectively-measured estimate, were reported. The prevalence of weight status is presented for normal, overweight and obese status, as well as for combined overweight and obese status.

Fourth, agreement between self-reported and objectively-measured height, weight, and BMI was assessed using Lin’s concordance correlation coefficient (rho_c) by (1) sex and (2) race/ethnicity for 8^th^- and 11^th^-grade students, separately. Because Lin’s concordance correlation coefficient combines measures of precision (Pearson correlation) and accuracy (bias correction factor=C-bias), the overall correlation as well as estimates reflecting accuracy and precision are reported.

Fifth, we developed correction equations to estimate objectively measured height, weight, and BMI (i.e., dependent variables) from self-reported height and/or weight by grade, sex, and race/ethnicity using weighted linear regression. Although, SPAN did not sample by age, age was included as a covariate in the initial correction equations. However, age was not a statistically significant contributor to the equations and was removed from the final correction equations. Because there were statistically significant differences noted by grade (2 levels), sex (2 levels), and race/ethnicity (3 levels), 12 (=2*2*3) linear correction equations for these combinations are reported. The coefficient of determination (R^2^) reported how much of the variability of the dependent variables was explained by the independent variables for each correction equation.

Sixth, the validity of BMI from self-reported height and weight data to appropriately classify overweight and obesity status was assessed by estimating the (1) sensitivity, (2) specificity and (3) positive predictive value by grade level and by sex. Finally, self-reported measures and correction equations were used to estimate weight status. Sensitivity, specificity and positive predictive values of the correction equations were computed.

## Results

## Demographics

As shown in Table 1, the mean age for 8^th^- and 11^th^-grade students was 13.7, and 16.7 years, respectively. These mean ages were consistent with the age based on grade level. Around 53% of the sample was male, and the majority (56.6%) were non-Hispanic White. Using the objective measurements, the median and standard error (SE) values for BMI were 21.5 kg/m^2^ (±0.18SE) and 23.1(±0.16SE) kg/m^2^ among 8^th^- and 11^th^-graders, respectively. Among 8^th^-grade students, the prevalence of overweight and obesity was 17.9% and 18.1%, respectively. The prevalence of overweight and obesity in 11^th^-grade students was 16.5% and 15.8%, respectively.

**Table 1:** Descriptive statistics of SPAN 2000-2002 and 2004-2005 by grade

Characteristic	2000-2002		2004-2005		All	
	8th (n=5362) N=(288584)	11th (n=3576) N=(249363)	8th (n=8827) N=(291672)	11th (n=6456) N=(233753)	8th (n=14189) N=(580256)	11th (n=10032) N=(483116)
Age, y, mean(SE)	13.7(0.03)	16.7(0.05)	13.7(0.04)	16.7(0.03)	13.7(0.02)	16.7(0.03)
Boys	54.6(3.05)	55.0(2.93)	50.8(1.26)	50.5(2.31)	52.7(1.60)	52.8(1.90)
Race/Ethnicity (%(SE))						
African American	11.2(2.92)	9.2(2.41)	14.7(3.02)	14.2(2.69)	13.0(2.13)	11.6(1.77)
Hispanic	40.9(4.09)	26.9(3.71)	41.7(4.27)	36.9(2.65)	41.3(3.09)	31.7(2.76)
White/Other	47.9(3.85)	63.9(4.80)	43.6(4.44)	48.8(3.78)	45.7(3.04)	56.6(3.40)
Self-Reported						
Height (cm) median(SE)	161.6(0.41)	169.4(0.72)	160.9(0.75)	166.7(0.47)	161.3(0.43)	168.4(0.53)
Weight (Kg) median(SE)	56.4(0.72)	65.8(0.93)	56.1(0.61)	65.2(0.74)	56.3(0.56)	65.6(0.61)
BMI (Kg/m^2^) median(SE)	21.1(0.26)	22.6(0.14)	21.2(0.21)	22.7(0.28)	21.1(0.16)	22.6(0.18)
Weight status (%)						
Normal	65.1(2.50)	72.7(3.57)	65.7(2.35)	67.7(1.56)	65.4(1.69)	70.3(2.20)
Overweight	19.5(2.52)	15.1(2.53)	20.0(1.68)	15.7(0.90)	19.7(1.48)	15.4(1.40)
Obese	15.4(1.54)	12.2(1.43)	14.3(1.21)	16.6(1.30)	14.8(0.95)	14.3(1.09)
Objectively-measured						
Height (cm) median(SE )	162.2(0.36)	169.5(0.49)	162.2(0.41)	167.3(0.46)	162.2(0.27)	168.6(0.45)
Weight (Kg) median(SE )	57.5(0.49)	66.4(0.78)	56.9(0.45)	65.1(1.08)	57.1(0.38)	66.1(0.71)
BMI (Kg/m^2^) median(SE )	21.4(0.36)	22.9(0.18)	21.6(0.20)	23.2(0.26)	21.5(0.18)	23.1(0.16)
Weight status (%)						
Normal	63.5(2.48)	68.8(3.90)	64.5(2.39)	66.4(1.98)	64.0(1.65)	67.6(2.26)
Overweight	17.8(2.17)	16.8(2.66)	18.0(1.38)	16.3(1.60)	17.9(1.26)	16.5(1.55)
Obese	18.7(1.69)	14.5(1.64)	17.5(1.62)	17.3(1.35)	18.1(1.14)	15.8(1.17)

n represents the sample size in the survey and N indicates the estimated population size using the sampling weights. SE: Standard error.

White/other category includes non-Hispanic White, Asian, Pacific Islander, Native American, and “other”

Using the U.S. Centers for Disease Control and Prevention (CDC) sex and age BMI growth charts, students are classified into underweight/normal (<85th percentile), overweight (≥85th percentile to <95th percentile) and obese (≥95th percentile) weight status categories.

## Differences in medians of height, weight, and BMI

With regards to height, among 8^th^-grade students, statistically significant differences between self-reported and objective measurements were seen in (1) Hispanic boys, (2) Hispanic girls and (3) African-American girls (Table 2). Across all categories of race/ethnicity, 11^th^-grade students overestimated their height, ranging from 0.68 to 1.04 cm among girls and from 1.87 to 2.02 cm among boys.

**Table 2:** Differences between self-reported and objective measures in SPAN 2000-2002 and 2004-2005 pooled data

	Difference in Height (cm)				Difference in Weight (kg)				Difference in BMI (kg/m^2^)			
	Girls		Boys		Girls		Boys		Girls		Boys	
	Median (95%CI)	p-value	Median (95%CI)	p-value	Median (95%CI)	p-value	Median (95%CI)	p-value	Median (95%CI)	p-value	Median (95%CI)	p-value
8th Grade												
African American	1.22 (0.69,1.75)	<0.001	0.92 (-0.62,2.46)	0.238	-0.98 (-1.62,-0.33)	0.003	-0.29 (-1.28,0.69)	0.555	-0.70 (-0.94,-0.45)	<0.001	-0.27 (-0.90,0.36)	0.394
Hispanic	0.78 (0.20,1.36)	0.008	0.80 (0.27,1.33)	0.003	-0.40 (-0.57,-0.24)	<0.001	-0.68 (-1.03,-0.33)	<0.001	-0.45 (-0.66,-0.25)	<0.001	-0.49 (-0.72,-0.26)	<0.001
White / Other	0.29 (-0.08,0.66)	0.119	-0.23 (-0.81,0.35)	0.437	-0.76 (-0.96,-0.57)	<0.001	-0.30 (-0.61,0.35)	0.053	-0.49 (-0.57,-0.41)	<0.001	-0.23 (-0.41,-0.04)	0.015
11th Grade												
African American	0.81 (0.13,1.50)	0.019	2.02 (1.42,2.61)	<0.001	-0.82 (-1.32,-0.31)	0.001	-0.41 (-0.92,0.09)	0.104	-0.51 (-0.90,-0.11)	0.012	-0.58 (-0.80,-0.35)	<0.001
Hispanic	1.04 (0.69,1.75)	0.001	1.87 (1.46,2.27)	<0.001	0.004 (-0.42,0.43)	0.986	0.08 (-0.18,0.33)	0.548	-0.26 (-0.50,-0.02)	0.032	-0.52 (-0.67,-0.36)	<0.001
White / Other	0.68 (0.42,1.67)	<0.001	1.87 (1.27,2.47)	<0.001	-0.45 (-0.80,-0.10)	0.011	0.39 (-0.05,0.82)	0.08	-0.49 (-0.64,-0.34)	<0.001	-0.39 (-0.59,-0.18)	<0.001

p-value associated with the median difference between self-reported and objective measures.

White/other category includes non-Hispanic White, Asian, Pacific Islander, Native American, and “other”

With regards to weight, among girls there were statistically significant differences between self-reported and objectively-measured estimates across all categories of race/ethnicity in both grades, with the exception of 11^th^-grade Hispanic girls. Hispanics boys in 8^th^-grade underreported their weight [median -0.68 kg 95% CI (-1.03; -0.33)]. With regard to BMI, estimates obtained from self-reported measurements were lower than those from objectively-measured data across all grade, sex and race/ethnicity categories, with the exception of 8^th^-grade African-American boys. Differences ranged from -0.23 to -0.70 kg/m^2^.

## Differences in prevalence of weight status

Overall self-reported height and weight data underestimated the prevalence of overweight and obesity when compared to the objective measures (Table 3). Further, the absolute and relative error estimates varied by grade and sex. The largest underestimation of the prevalence of obesity was shown among 8^th^-grade girls (absolute and relative error of -4.1% and -0.25, respectively).

**Table 3:** Prevalence of weight status categories (Standard error of percent), absolute error and relative error

Weight Status	Self-reported prevalence (%)			Directly measured prevalence(%)			Absolute Error(%)			Relative Error		
	Girls	Boys	All	Girls	Boys	All	Girls	Boys	All	Girls	Boys	All
8th Grade												
Normal	69.9(2.33)	61.5(1.97)	65.5(1.69)	65.1(2.01)	63.0(2.21)	64.0(1.65)	4.8	-1.5	1.5	0.07	-0.02	0.02
Overweight	18.0(2.06)	21.2(1.67)	19.7(1.48)	18.8(1.73)	17.1(1.78)	17.9(1.26)	-0.8	4.1	1.8	-0.04	0.24	0.10
Obese	12.1(0.71)	17.3(1.45)	14.8(0.95)	16.1(1.15)	19.9(1.47)	18.1(1.14)	-4.0	-2.6	-3.3	-0.25	-0.13	-0.18
Overweight/Obese	30.1(2.33)	38.5(1.97)	34.5(1.69)	34.9(2.01)	37.0(2.21)	36.0(1.65)	-4.8	1.5	-1.5	-0.14	0.04	0.04
11th Grade												
Normal	76.0(1.76)	65.2(3.09)	70.3(2.20)	72.6(2.21)	63.2(2.82)	67.6(2.26)	3.4	2.0	2.7	0.05	0.03	0.04
Overweight	14.0(1.34)	16.6(1.85)	15.4(1.40)	15.7(1.61)	17.3(1.88)	16.5(1.55)	-1.7	-0.7	-1.1	-0.11	-0.04	-0.07
Obese	10.0(0.97)	18.2(1.77)	14.3(1.09)	11.7(1.16)	19.5(1.70)	15.9(1.17)	-1.7	-1.3	-1.6	-0.15	-0.07	-0.10
Overweight/Obese	24.0(1.76)	34.8(3.09)	29.7(2.20)	27.4(2.21)	36.8(2.82)	32.4(2.26)	-3.4	-2.0	-2.7	-0.12	-0.05	-0.08

Using the U.S. Centers for Disease Control and Prevention (CDC) sex and age BMI growth charts, students are classified as underweight/normal (<85th percentile), overweight (≥85th percentile to <95th percentile) and obese (≥95th percentile) weight status categories.

Absolute error: Self-reported prevalence minus objectively-measured prevalence. Difference between error values given and error values calculated using prevalence in table are due to rounding of prevalence estimates.

Relative error: Absolute error divided by objectively-measured prevalence.

## Concordance coefficients

Table 4 shows (1) Lin’s concordance coefficients, (2) precision (Pearson correlation) and (3) accuracy (bias-correction factor), between self-reported and objectively-measured height, weight, and BMI for 8^th^- and 11^th^-grade students. Lin’s concordance coefficients for height ranged from 0.60 to 0.90; for weight from 0.82 to 097; and for BMI, from 0.78 to 0.95. The lowest precision was found in height among 8^th^- and 11^th^-grade Hispanic girls (0.66) and the greatest precision observed was in weight among 11^th^-grade African-American boys (0.97).

**Table 4:** Lin’s concordance coefficients(95%CI), Pearson correlation coefficient and bias correction factor(BCF) between self-reported and objective measurements

8th-grade	Girls				Boys			
	LCC(95%CI)	p-value	Pearson	BCF	LCC(95%CI)	p-value	Pearson	BCF
Height								
African American	0.70 (0.69,0.70)	<0.001	0.76	0.92	0.86 (0.86,0.86)	<0.001	0.90	0.96
Hispanic	0.60 (0.59,0.60)	<0.001	0.66	0.91	0.64 (0.63,0.64)	<0.001	0.69	0.92
White/Other	0.82 (0.82,0.82)	<0.001	0.84	0.98	0.85 (0.84,0.85)	<0.001	0.86	0.98
All	0.73 (0.72,0.73)	<0.001	0.76	0.96	0.77 (0.77,0.77)	<0.001	0.80	0.96
Weight								
African American	0.82 (0.81,0.82)	<0.001	0.83	0.99	0.95 (0.95,0.95)	<0.001	0.95	1.00
Hispanic	0.90 (0.89,0.90)	<0.001	0.91	0.99	0.92 (0.92,0.92)	<0.001	0.93	0.99
White/Other	0.92 (0.92,0.92)	<0.001	0.93	0.99	0.94 (0.93,0.94)	<0.001	0.94	1.00
All	0.90 (0.89,0.90)	<0.001	0.90	0.99	0.93 (0.93,0.93)	<0.001	0.94	0.99
BMI								
African American	0.78 (0.78,0.79)	<0.001	0.80	0.98	0.90 (0.90,0.90)	<0.001	0.91	0.99
Hispanic	0.81 (0.81,0.81)	<0.001	0.82	0.99	0.83 (0.83,0.83)	<0.001	0.84	0.99
White/Other	0.86 (0.86,0.87)	<0.001	0.88	0.98	0.86 (0.86,0.86)	<0.001	0.86	1.00
All	0.83 (0.83,0.83)	<0.001	0.84	0.99	0.85 (0.85,0.85)	<0.001	0.86	1.00
11th Grade	Girls				Boys			
	LCC(95%CI)	p-value	Pearson	BCF	LCC((95%CI)	p-value	Pearson	BCF
Height								
African American	0.82 (0.82,0.82)	<0.001	0.85	0.97	0.90 (0.90,0.91)	<0.001	0.95	0.95
Hispanic	0.61 (0.61,0.62)	<0.001	0.66	0.92	0.71 (0.71,0.71)	<0.001	0.76	0.94
White/Other	0.89 (0.89,0.89)	<0.001	0.90	0.98	0.89 (0.89,0.89)	<0.001	0.92	0.96
All	0.80 (0.80,0.81)	<0.001	0.83	0.97	0.84 (0.84,0.84)	<0.001	0.87	0.96
Weight								
African American	0.92 (0.91,0.92)	<0.001	0.92	1.00	0.96 (0.96,0.96)	<0.001	0.97	0.99
Hispanic	0.96 (0.96,0.96)	<0.001	0.96	1.00	0.95 (0.95,0.95)	<0.001	0.95	1.00
White/Other	0.96 (0.96,0.96)	<0.001	0.96	1.00	0.94 (0.94,0.94)	<0.001	0.94	1.00
All	0.95 (0.95,0.95)	<0.001	0.95	1.00	0.95 (0.95,0.95)	<0.001	0.95	1.00
BMI								
African American	0.86 (0.86,0.86)	<0.001	0.87	0.99	0.93 (0.93,0.93)	<0.001	0.95	0.98
Hispanic	0.88 (0.88,0.88)	<0.001	0.88	0.99	0.84 (0.84,0.85)	<0.001	0.85	1.00
White/Other	0.92 (0.92,0.92)	<0.001	0.93	0.99	0.91 (0.91,0.91)	<0.001	0.92	0.99
All	0.89 (0.89,0.89)	<0.001	0.90	0.99	0.89 (0.89,0.89)	<0.001	0.90	0.99

Lin’s concordance coefficient=LCC

White/other category includes non-Hispanic White, Asian, Pacific Islander, Native American, and “other”

p-values associated with testing the null hypothesis that the Lin’s concordance coefficient is equal to zero.

## Linear regression equations

The lowest amount of variation in objectively-measured height explained by self-reported height was observed among Hispanics, regardless of their sex (43.1% for 8^th^-grade girls, 47.4% for 8^th^-grade boys, 44% for 11^th^-grade girls and 56.9% for 11^th^-grade boys) (Table 5). This indicates that there are some additional factors that were not measured in SPAN to predict objectively-measured height. The variation of objective measures explained by self-reported measures was higher for weight than for height for all sex, grade, and across race/ethnicity categories (Table 6). Across sex, grade, and race/ethnicity, the BMI variability explained when using self-reported weight and the square inverse of self-reported height was above 77% with the exception of 8^th^-grade African-American girls (65.9%) (see Table 7)

**Table 5:** Correction equations to estimate objectively-measured height (cm) using self-reported data

	Girls			Boys		
	n (N)	Correction Equation	R^2^	n (N)	Correction Equation	R^2^
Grade=8						
African American	838 (35248)	72.93+0.54 SR_Height_	57.1%	723 (38329)	53.18+0.68 SR_Height_	80.4%
Hispanic	2998 (102957)	89.41+0.43 SR_Height_	43.1%	2758 (124170)	88.40+0.46 SR_Height_	47.4%
White/Other	2991 (126109)	48.94+0.69 SR_Height_	70.0%	3250 (134664)	48.77+0.71 SR_Height_	74.3%
Grade=11						
African American	619 (28620)	54.42+0.66 SR_Height_	72.0%	502 (26750)	34.32+0.80 SR_Height_	89.6%
Hispanic	1826 (70726)	80.92+0.48 SR_Height_	44.0%	1695 (78070)	74.52+0.56 SR_Height_	56.9%
White/Other	2532 (123589)	31.84+0.80 SR_Height_	81.8%	2647 (145409)	27.75+0.83 SR_Height_	85.5%

Grade 8 students had a mean age of 13.7 years old (SE=0.02) and their percentiles 25% and 75% are 12.7 and 13.7 years old, respectively

White/other category includes non-Hispanic White, Asian, Pacific Islander, Native American, and “other”

Grade 11 students had a mean age of 16.7 years old (SE=0.03) and their percentiles 25% and 75% are 15.7 and 16.7 years old, respectively

n = sample size; N = estimated population size using the sampling weights, R^2^ = coefficient of determination in percentage; SR = self-reported

**Table 6:** Correction equations to estimate objectively-measured weight (Kg) using self-reported data

	Girls			Boys		
	n (N)	Correction Equation	R^2^	n (N)	Correction Equation	R^2^
Grade=8						
African American	832 (35290)	11.30+0.85 SR_Weight_	68.1%	720 (38025)	3.44+0.96 SR_Weight_	90.9%
Hispanic	3058 (104284)	-0.04+1.02 SR_Weight_	82.0%	2817 (124777)	0.46+1.02 SR_Weight_	86.1%
White/Other	2992 (125746)	1.35+1.00 SR_Weight_	86.5%	3269 (136071)	1.41+0.99 SR_Weight_	88.2%
Grade=11						
African American	608 (28565)	6.24+0.93 SR_Weight_	84.7%	506 (26932)	-5.21+1.08 SR_Weight_	93.1%
Hispanic	1847 (71924)	-1.28+1.03 SR_Weight_	91.9%	1714 (78732)	1.18+0.99 SR_Weight_	90.0%
White/Other	2520 (122353)	-0.34+1.02 SR_Weight_	92.9%	2652 (146334)	2.07+0.97 SR_Weight_	88.4%

Grade 8 students had a mean age of 13.7 years old (SE=0.02) and their percentiles 25% and 75% are 12.7 and 13.7 years old, respectively

White/other category includes non-Hispanic White, Asian, Pacific Islander, Native American, and “other”

Grade 11 students had a mean age of 16.7 years old (SE=0.03) and their percentiles 25% and 75% are 15.7 and 16.7 years old, respectively

n = sample size; N = estimated population size using the sampling weights, R^2^ = coefficient of determination in percentage; SR = self-reported

**Table 7:** Correction equations to estimate objectively-measured BMI (kg/m^2^) using self-reported data

	Girls			Boys		
	n (N)	Correction Equation	R^2^	n (N)	Correction Equation	R^2^
Grade=8						
African American	814 (34567)	-5.94+0.32 SR_Weight_+27.31(1/SR_Height_)^2^	65.9%	697 (37688)	-12.82+0.32 SR_Weight_+43.58(1/SR_Height_)^2^	83.8%
Hispanic	2879 (98951)	-5.60+0.39 SR_Weight_+17.36(1/SR_Height_)^2^	77.8%	2697 (121516)	-6.11+0.34 SR_Weight_+22.24(1/SR_Height_)^2^>	79.0%
White/Other	2945 (124790)	-8.21+0.36 SR_Weight_+26.50(1/SR_Height_)^2^	80.7%	3206 (133815)	-10.71+0.32 SR_Weight_+36.18(1/SR_Height_)^2^	78.1%
Grade=11						
African American	603 (28479)	-10.46+0.34 SR_Weight_+35.83(1/SR_Height_)^2^	79.6%	494 (26523)	-20.95+0.35 SR_Weight_+59.86(1/SR_Height_)^2^	90.9%
Hispanic	1795 (70041)	-5.88+0.36 SR_Weight_+20.75(1/SR_Height_)^2^	83.3%	1671 (77409)	-3.18+0.29 SR_Weight_+20.51(1/SR_Height_)^2^	80.4%
White/Other	2498 (120375)	-15.41+0.37 SR_Weight_+43.15(1/SR_Height_)^2^	87.9%	2618 (143966)	-16.78+0.31 SR_Weight_+55.83(1/SR_Height_)^2^	84.7%

Grade 8 students had a mean age of 13.7 years old (SE=0.02) and their percentiles 25% and 75% are 12.7 and 13.7 years old, respectively

White/other category includes non-Hispanic White, Asian, Pacific Islander, Native American, and “other”

Grade 11 students had a mean age of 16.7 years old (SE=0.03) and their percentiles 25% and 75% are 15.7 and 16.7 years old, respectively

n = sample size; N = estimated population size using the sampling weights, R^2^ = coefficient of determination in percentage; SR = self-reported

## Sensitivity, specificity and positive predictive value

As shown in Table 8, sensitivity, which is the proportion of overweight students correctly classified by self-reported weight status, was 60.5% and 62.0% for 8^th^- and 11^th^-grade students, respectively. The sensitivity for obese weight status was 70.8% and 81.9% for 8^th^- and 11^th^-grade students, respectively. The sensitivity for combined overweight/obese status was 81.3% and 83.1% for 8^th^- and 11^th^-grade students, respectively. Further, specificity, which is the proportion of students who are not overweight/obese who are classified as not overweight/obese by self-reported weight and height was 91.1% and 95.6% for 8^th^- and 11^th^-grade students, respectively. The specificity for obese weight status was 97.0% and 98.1% for 8^th^- and 11^th^-grade students, respectively.

**Table 8:** Sensitivity, specificity and positive predictive value of self-reported data and correction equations for weight status

Grade	Weight Status	Sensitivity			Specificity			Positive Predictive Value		
		Girls	Boys	All	Girls	Boys	All	Girls	Boys	All
Self-Reported										
8th	Overweight	58.2%	62.7%	60.5%	91.2%	87.5%	89.2%	60.1%	51.6%	55.3%
	Obese	66.8%	73.6%	70.8%	97.7%	96.4%	97.0%	84.0%	83.0%	83.4%
	Overweight/Obese	77.2%	84.6%	81.3%	93.9%	88.5%	91.1%	86.7%	81.2%	83.4%
11th	Overweight	64.1%	60.4%	62.0%	95.2%	92.7%	93.9%	71.1%	63.8%	66.9%
	Obese	76.3%	85.0%	81.9%	98.9%	97.5%	98.1%	89.8%	88.7%	89.1%
	Overweight/Obese	81.4%	84.2%	83.1%	97.5%	93.6%	95.6%	92.5%	88.4%	89.9%
Correction Equations										
8th	Overweight	67.4%	62.5%	64.8%	86.8%	86.9%	86.9%	54.0%	50.2%	52.1%
	Obese	73.4%	80.2%	77.4%	97.1%	96.5%	96.8%	82.0%	84.7%	83.7%
	Overweight/Obese	86.0%	86.3%	86.1%	88.2%	86.9%	87.5%	78.9%	79.4%	79.2%
11th	Overweight	72.0%	69.6%	70.7%	91.2%	90.6%	90.9%	59.9%	61.2%	60.6%
	Obese	82.4%	85.5%	84.4%	99.0%	97.2%	98.1%	91.4%	87.8%	89.0%
	Overweight/Obese	85.9%	89.7%	88.2%	92.0%	91.1%	91.5%	80.1%	85.2%	83.2%

Using the U.S. Centers for Disease Control and Prevention (CDC) sex and age BMI growth charts, we classified students into underweight/normal (<85th percentile), overweight (≥85th percentile to <95th percentile) and obese (≥95th percentile) weight status categories

Sensitivity: proportion of the weight status category correctly classified by self-reported measurements in that weight status category

Specificity: proportion of students who were not in the weigh status category as correctly classified as not in that weight status category by self-reported measurements.

Positive predictive value: proportion of students identified by self-reported measures with a particular weight status that are truly in such particular weight status

The positive predictive value, which is the proportion of students identified by self-reported weight status as overweight/obese that are truly overweight/obese was 83.4% and 89.9% among 8^th^- and 11^th^-grade students, respectively; the positive predictive value for obese as weight status was 83.4% and 89.1% among 8^th^- and 11^th^-grade students, respectively.

The sensitivity of estimates obtained using the correction equations was improved over the self-reported measures. However, the positive predictive value was not consistently improved across sex, grades and weight status (Table 8).

## Discussion

This study examined the bias, precision and accuracy of self-reported height, weight, and BMI derived from self-reported measures, across all sex, grade and race/ethnicity using data from a large surveillance system of 8th- and 11th-grade Texas adolescents. Due to the lack of prior studies exploring these properties in adolescents using probability samples, it is difficult to discuss our findings within the context of previous research. Despite this, we identified several findings. Prior studies have recommended against the use of self-report data from children under the age of 14 [4,6,17,18]. This study suggests that a slightly lower age cutoff for using self-reported height and weight may be appropriate, but this is arguable. Although statistically significant differences were noted between self-report and objective measures overall and by subpopulations, median differences between self-report and objective measures were small. Overall, height tended to be over-reported relative to objective measures. Self-reported measurements were taken in feet and inches; children are unlikely to report height with a greater precision than 0.5 inches. The increased bias in height and a decreased bias in weight resulted in a small downward bias in BMI calculated from self-report measures in almost all sex, grade, and the race/ethnicity subpopulations. These findings are generally consistent with previous publications [5-7,10,14,16,19-22,36]. The slightly lower relative errors in estimating the prevalence of overweight/obese among youth in Texas could be due to the fact that, since 1998, it is standard practice to have height and weight measured during sports team participation or physical education classes in schools and could potentially create greater awareness of actual height and weight in our population [37]. This could also explain why Lin’s concordance coefficients were similar for height and weight for this Texas sample compared to samples from nationally representative data in the U.S. [6]. These sensitivity results in Texas are similar to results from nationally representative data (range 59% to 76% for overweight/obese and 70.2% to 74% for obese) [26].

To our knowledge, this is the first study to develop correction equations to estimate measured height, weight and BMI from self-reported data by each level of sex (two levels), grade (two levels), and race/ethnicity (three levels) among adolescents. With correction equations, sensitivity to classify overweight/obese and obese status rose to a minimum of 85.9% and 76.3%, respectively, across categories. These equations are particularly useful for public health researchers who are restricted to self-reported measures of height and weight due to budget and/or staff resource constraints. However, the positive predictive value of weight status was not improved by the correction equations. Researchers interested in obesity as an outcome will need to weigh the pros and cons of using correction equations instead of self-reported measures within the context of their own research and study design.

This study has several strengths and limitations. One of the strengths is that Texas can be viewed as a representative state in terms of its increasingly diverse racial/ethnic profile and the steadily increasing Hispanic population [38]. Thus, the findings of this study will be applicable to the projected changes in U.S. racial/ethnic demography [39]. The second strength is the large sample size and availability of a representative sample of middle school and high school students in Texas, both of which greatly contribute to the generalizability of these findings. One limitation is the study’s need to collapse non-Hispanic White, Asian, Pacific Islander, Native American, and “other” within the White/other category. Retaining the original categories will require a large sample size. Asian girls usually have lower BMI than other girls and we were unable to capture this in SPAN. As previously mentioned, grade level, rather than age was used in the sampling strategy. While this limits the interpretation of results to an international audience, grade provides a reasonable proxy for age. Finally, menarche can also change BMI as fat distribution changes, but SPAN did not measure age of menarche to account for this. One final limitation is that data on the school-level percentage of students receiving free and reduced lunch, a marker of socio-economic status, were unavailable for the 2000–2002 academic year and, therefore, not included as a term in the correction equations.

## Conclusions

Direct measurements of height and weight are preferable over self-report measures when seeking to estimate prevalence of weight status among students. However, when direct measurement is not practical, self-reported measurements provide a reasonable proxy measure across grade, sex, and racial/ethnic subpopulations of adolescents. Researchers should be cautious of the potential for bias, particularly among girls. This study’s findings suggest that the use of correction equations for reported data is a reasonable alternative when direct measurement of height and weight is not feasible.

## Competing interests

The authors declare that they do not have competing interests.

## Authors’ contributions

AP contributed to this manuscript by originating the idea of the paper, participating in the literature review, generate the data analysis plan, generated the statistical analysis, interpretation of the data and primary authorship of manuscript, including reviewing co-authors contributions, revisions and approval of the final version of the manuscript. AP is prepared to take public responsibility for the results in the article. KPG participated in the concept and design of the paper, interpretation of results, and significant review of the manuscript. EKN contributed to this manuscript by participating in the concept development and literature review and contributed to the drafting of the manuscript. DJM participated in critical review of the manuscript. DMH obtained funding, served as principal investigator of the parent study, developed the design of the overall parent study, directed data collection, and participated in critical review of the manuscript. All authors read and approved the final manuscript.

## Declarations

This article has been published as part of *International Journal of Behavioral Nutrition and Physical Activity* Volume 12 Supplement 1, 2015: The science of childhood obesity: an individual to societal framework. The full contents of the supplement are available online at http://www.ijbnpa.org/supplements/12/S1. Publication costs for this supplement were funded by the Michael & Susan Dell Foundation.
